# Robotic-Assisted Surgery in Children Using the Senhance^®^ Surgical System: An Observational Study

**DOI:** 10.3390/children11080935

**Published:** 2024-07-31

**Authors:** Rianne E. M. Killaars, Ruben G. J. Visschers, Marc Dirix, Olivier P. F. Theeuws, Roxanne Eurlings, Dianne J. H. Dinjens, Hamit Cakir, Wim G. van Gemert

**Affiliations:** 1 Department of Pediatric Surgery, MosaKids Children’s Hospital, Maastricht University Medical Center+ (MUMC+), P. Debyelaan 25, 6229 HX Maastricht, The Netherlands; 2 European Consortium of Pediatric Surgery (MUMC+, Uniklinik Aachen, Centre Hospitalier Chrétien Liège), Maastricht, P. Debyelaan 25, 6229 HX Maastricht, The Netherlands; 3 Research Institute of Nutrition and Translational Research in Metabolism NUTRIM, Universiteitssingel 40, 6229 ER Maastricht, The Netherlands

**Keywords:** pediatric patients, children, robotic-assisted surgery, Senhance Surgical System, robotic-assisted laparoscopy, minimally invasive surgery

## Abstract

Background: Robotic-assisted surgery (RAS) holds many theoretical advantages, especially in pediatric surgical procedures. However, most robotic systems are dedicated to adult surgery and are less suitable for smaller children. The Senhance^®^ Surgical System (SSS^®^), providing 3 mm and 5 mm instruments, focuses on making RAS technically feasible for smaller children. This prospective observational study aims to assess whether RAS in pediatric patients using the SSS^®^ is safe and feasible. Methods and Results: A total of 42 children (aged 0–17 years, weight ≥ 10 kg) underwent a RAS procedure on the abdominal area using the SSS^®^ between 2020 and 2023. The study group consisted of 20 male and 22 female individuals. The mean age was 10.7 years (range 0.8 to 17.8 years), with a mean body weight of 40.7 kg (range 10.1 to 117.3 kg). The 3-mm-sized instruments of the SSS^®^ were used in 12 of the 42 children who underwent RAS. The RAS procedures were successfully completed in 90% of cases. The conversion rate to conventional laparoscopy was low (10%), and there were no conversions to open surgery. One of the 42 cases (2%) experienced intraoperative complications, whereas six children (14%) suffered from a postoperative complication. Overall, 86% of the patients had an uncomplicated postoperative course. Conclusions: The results of the current observational study demonstrate the safety and feasibility of utilizing the SSS^®^ for abdominal pediatric RAS procedures. The study provides new fundamental information supporting the implementation of the SSS^®^ in clinical practice in pediatric surgery.

## 1. Introduction

In recent years, minimally invasive surgery (MIS) has been fully embraced by surgeons and their patients in the adult population. MIS has demonstrated remarkable advancements, constantly pushing the boundaries of surgical techniques for better surgical benefits. Anatomical challenges unique to children, which require the adaptation of materials to suit smaller patients, have delayed the evolution of MIS within pediatric surgery. Nevertheless, laparoscopy has demonstrated its benefits over the open approach and, even in most pediatric cases, replaced it as the gold standard [1,2]. Laparoscopic surgery results in better cosmesis and significantly accelerates patients’ recovery in terms of reduced postoperative pain, complications, and lengths of hospital stay [1,3]. However, conventional laparoscopy still has recognizable limitations, such as a two-dimensional view that limits the depth perception within the surgical field, restricted instrument movements, and non-ergonomically comfortable positions for the surgeon [4].

Robotic-assisted surgery (RAS) is a further advancement over the minimally invasive technique, in which the instruments are remotely controlled by the surgeon at a console alongside the patient. RAS provides significant benefits, including stable two- or three-dimensional vision, an increased range of motion, motion scaling and tremor reduction, intuitive movement, and optimized comfort for the operating surgeon [4,5,6,7,8]. This possibly addresses laparoscopic difficulties and enhances surgeons’ performance of their surgical procedures. RAS may enhance the operative precision, which subsequently reduces the trauma to the surrounding tissue. Combined with small abdominal incisions, this may result in reduced pain after surgery and a shorter postoperative recovery time [6,7,8].

In adults, RAS is widely accepted and used within the surgical field. However, its implementation in pediatric surgery is progressing slowly. The restricted internal and external operating areas and variations in body size and proportionality, along with delicate anatomical structures, tend to be challenging when performing RAS in children. The trocar and patient positions must be adapted based on the child’s age, body size, and proportions. Nevertheless, the proper positioning of trocars is important as the abdominal wall is thin in children [4]. Therefore, in small children and neonates, 3 mm instruments are mostly used in laparoscopic procedures.

Despite showing promising results in pediatric patients, the Da Vinci^®^ Surgical System (DVSS^®^) appears to be limited in smaller workspaces [9,10]. Therefore, the DVSS^®^, supplied with 8 mm instruments, is mainly used in adolescents [9,10].

The Senhance^®^ Surgical System (SSS^®^) focuses on making RAS technically feasible for even the smallest pediatric patients [6]. The SSS^®^ provides 3 mm instruments and introduces more specialized instruments for children. Features such as haptic feedback, a digital fulcrum, eye-tracking camera control, and independent robot arms enabling repositioning allow for better accuracy and safety in pediatric procedures. New and future innovations pave the way for RAS in young children and even neonates. Pediatric surgical procedures rely on surgical precision. The implementation of a more precise operative technique is expected to improve the outcomes.

The SSS^®^ has already demonstrated the ability to perform intracorporal suturing and knot tying in small cavities [11]. Until now, due to its relatively recent emergence on the market, clinical studies about the performance of the SSS^®^ in pediatric patients are limited. In September 2020, the world’s first pediatric robotic procedures using the SSS^®^ were performed at the Department of Pediatric Surgery at the MosaKids Children’s Hospital of the Maastricht University Medical Center (MUMC+) [12,13]. This study aims to evaluate the safety and feasibility of pediatric RAS procedures utilizing the SSS^®^, primarily evaluating the complication and conversion rates.

## 2. Materials and Methods

### 2.1. Research Setting

This was a prospective observational study on RAS using the SSS^®^ as per its labeled EU indication for pediatric use (weight equal to or above 10 kg, suitable for a conventional endoscopic technique) and procedures performed as per clinical practice [14]. The study was conducted at the MosaKids Children’s Hospital of the Maastricht University Medical Center (MUMC+), based in the Netherlands. All children aged 0 to 17 years, weighing ≥ 10 kg, who underwent RAS on the abdominal area using SSS^®^, were included during an enrollment period of two years. Informed consent for robotic assistance with prospective data acquisition and analysis was obtained from all parents and/or pediatric patients. The study was approved by the Medical Ethical Commission.

### 2.2. Subjects

All consecutive pediatric patients who consulted a pediatric surgeon at the MUMC+ between September 2020 and January 2023 and were electively scheduled for a laparoscopic operation in the abdominal area were considered for enrollment in the study. Of these, children under the age of 18, with a minimum weight of 10 kg as per the Senhance EU indications [14], and having obtained informed consent, were consecutively included in the study.

Children whose parents preferred to proceed with conventional laparoscopic surgery, or parents or children with an insufficient understanding of the Dutch language, were excluded from the study. Other exclusion criteria were as follows: children requiring surgery of the heart or greater vessels, children for whom a laparoscopic approach and endoscopic approach in the thoracic area was not appropriate, children with anesthetic contraindications, children with pacemakers or other implants with which electro-surgery must be avoided, children with cancer, patients pregnant over the second trimester of pregnancy, and the presence of any relevant severe condition or clinically relevant abnormal laboratory parameters that, in the opinion of the investigator, may have interfered with their participation in the study.

All patients were followed up over time, consisting of five contact moments as part of the standard clinical care. Patients were provided with a reflection period of at least one week before deciding to participate in the study.

### 2.3. Data and Clinical Outcomes

The data for the analysis were obtained during the RAS procedures. The collected data included patient demographics, along with intraoperative and postoperative parameters related to the utilization of the robot and the recovery of the children. Complications were classified using the Clavien–Dindo Classification [15]. To assess the comfort levels of the surgeons while performing RAS using the SSS^®^, questionnaires on the physical demand were obtained from the pediatric surgeons directly after surgery in half of the randomly selected cases. The survey (modified from the Surgery Task Load Index (SURG-TLX) consisted of a binary question asking if the surgeon had any physical discomfort, pain, or fatigue in specific body areas, followed by a rating scale from 1 to 10 if the response was affirmative [16].

The data reflected the pediatric surgeons’ first performance of RAS procedures with the use of the SSS^®^. The effectiveness of RAS in children using the SSS^®^ was assessed through the analysis of the primary outcome of the rate of unplanned conversion from RAS to an open approach during the surgical procedure. Secondary outcomes were unplanned conversion to conventional laparoscopy, the reasons for conversion, the total operative time, the docking time, and the postoperative hospital stay. The safety of RAS in children using the SSS^®^ was assessed through the analysis of intraoperative complications and postoperative complications (within 30 days of the procedure).

### 2.4. Senhance Surgical System (SSS^®^)

**Figure 1:**
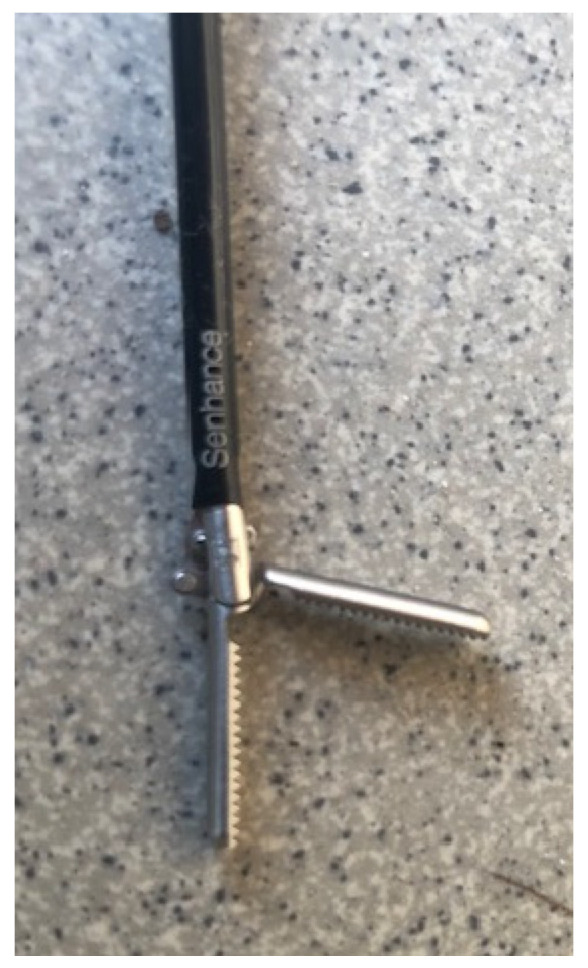
A 3 mm instrument from the Senhance Surgical System^®^.

The SSS^®^ with digital laparoscopy encompasses three main components. (1) The cockpit—the unsterile surgical console provides open communication with the operation field and features an ergonomic chair. The movements of the arms in the surgical field are directly manipulated through the hand and eye movements of the surgeon. (2) The manipulator arms—three independent robotic arms, each sterilely packed, strategically positioned at three different sides around the patient according to the trocar positions. The arm employs adapters to interface with the endoscope and surgical instruments during procedures. (3) The node—a relay unit, connecting the input of the surgeon to the manipulator arms and transmitting the video signals to the 2D/3D monitor in the cockpit. The system features are (1) robotic precision; (2) haptic sensing; (3) eye-tracking 3D camera control; (4) improved ergonomics; (5) an open-platform architecture—allowing direct communication between the surgeon and staff within the sterile field; (6) 3 mm (Figure 1) and 5 mm reusable instruments—also used in traditional laparoscopy; and (7) a digital fulcrum. The 3 mm instruments were utilized in younger children, while the 5 mm instruments were employed in older children. The use of the SSS^®^ is indicated for patients with a weight equal to or above 10 kg and a BMI of up to 40, who are suitable to undergo a conventional laparoscopic procedure.

The surgical team of each RAS procedure involved two pediatric surgeons: the first surgeon operated unsterile at the surgical console and the second surgeon assisted in a sterile position. Three surgeons rotated in performing the RAS procedures. The surgical team was reinforced by an operating room assistant and a second assisting surgeon in training at the surgical table.

A technical specialist from Asensus^®^ Surgical Inc. was on hand to provide guidance and advice on robotic use during the procedures. The three pediatric surgeons, all experienced in laparoscopic procedures, alternately performed the procedures. All members of the team had completed training on the SSS^®^ in both dry and wet labs. The first robotic procedure was performed in September 2020, when the SSS^®^ was introduced at the Department of Pediatric Surgery at the MUMC+.

### 2.5. Port Placement and Docking of the System

**Figure 2:**
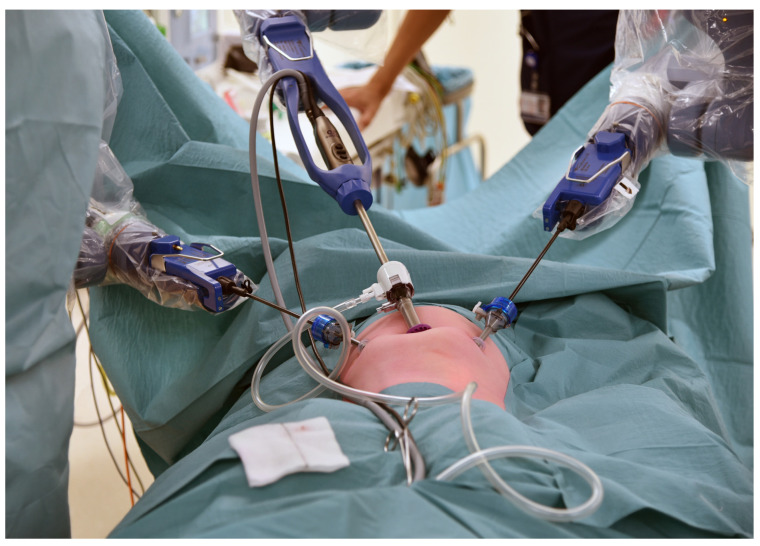
An example of the trocar positions during an inguinal hernia repair.

**Figure 3:**
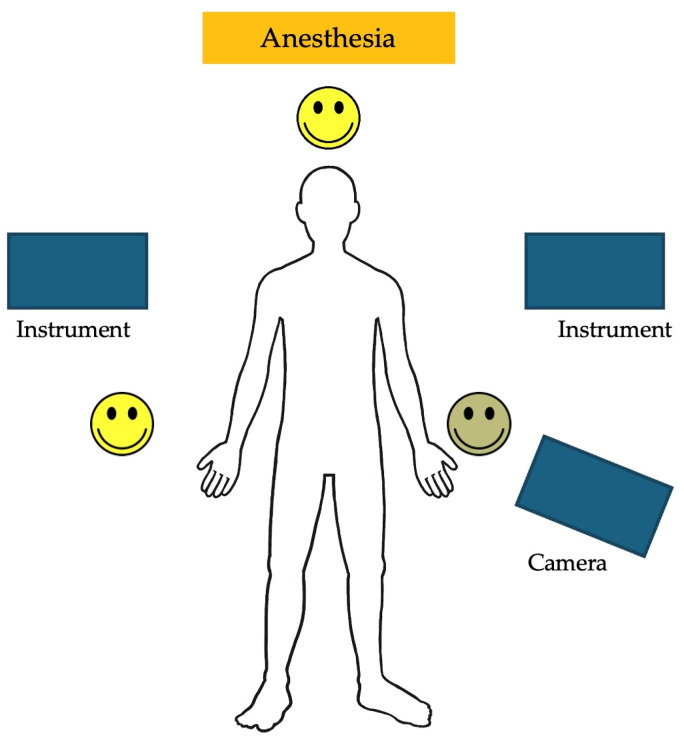
An example of the robotic arm positions during a robotic-assisted Nissen fundoplication.

The trocar positions and port placements were adapted according to the specific procedure, the patient’s size, and the workspace volume. See Figure 2 for the trocar positions during an inguinal hernia repair in a child. The setup of the SSS^®^ ensures that the trocar positions are similar to those in conventional laparoscopy and can be utilized accordingly. Care should be used to maximize the inter-trocar distance to minimize the possibility of collision of the robotic manipulator arms. After trocar insertion, the three independent surgical robotic arms are positioned strategically at three different sides around the patient (Figure 3).

### 2.6. Statistical Analysis

Descriptive statistics were applied to all variables. To check for a normal distribution, the Shapiro–Wilk test was used along with the visual inspection of histograms and normal Q–Q plots. A trend graph was created to provide an overview of the docking times over the study period. To assess whether there was a statistically significant difference in the average docking time and operative time between the initial and subsequent study periods, a paired *t*-test was conducted. A boxplot was used to illustrate the difference in the average docking time between these periods. Statistical significance was set at a *p*-value of less than 0.05. The analysis was carried out using IBM Statistical Product and Service Solutions (SPSS) Statistics 28.0.

## 3. Results

### 3.1. Characteristics of the Pediatric Patients

Between September 2020 and January 2023, a total of 42 pediatric patients (aged 0 to 17 years, weight > 10 kg) underwent an RAS procedure using the SSS^®^. All 42 pediatric patients were included in the study, completed the follow-up, and had their data successfully collected.

**Figure 4:**
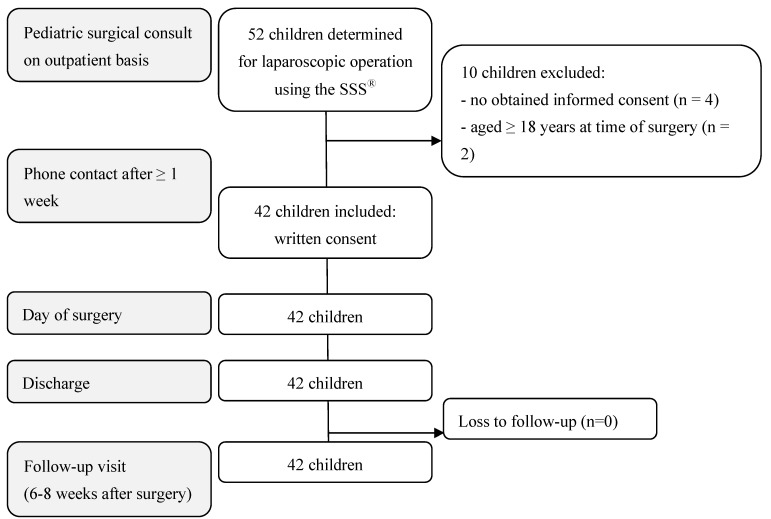
Flow chart of participants’ enrollment, inclusion, and follow-up.

Of the 52 pediatric patients determined for a laparoscopic operation using the SSS^®^, 42 children met the inclusion criteria. The reasons for exclusion were as follows: no obtained informed consent (4), age ≥ 18 years at the time of surgery (2), or body weight < 10 kg (4) (Figure 3 and Figure 4). The 42 RAS procedures performed using the SSS^®^ are listed in Table 1. The 3 mm instruments were used in twelve of the 42 RAS procedures (Table 1).

The study group consisted of 20 male and 22 female patients. The ages ranged from 0.8 to 17.8 years, with a mean age of 10.7 years. The mean body weight was 40.7 kg (range 10.1 to 117.3 kg) (Table 2).

### 3.2. Intraoperative Outcomes

**Figure 5:**
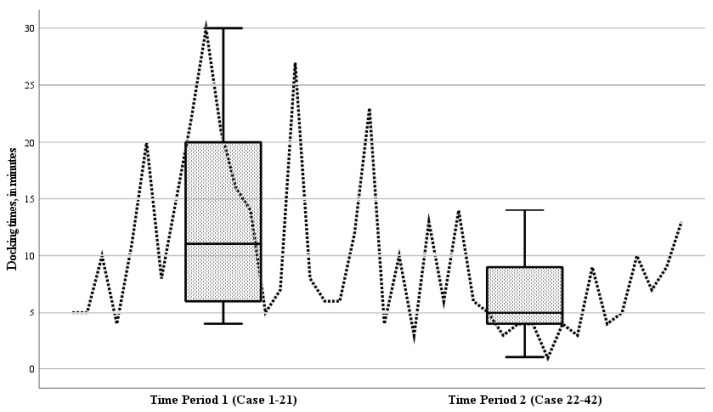
Boxplot of the docking times of the robot during the 42 robotic-assisted surgery (RAS) cases, for time period 1 (cases 1–21) and time period 2 (cases 22–42). Analyzing the docking times for individual cases over time revealed a decreasing trend; the mean docking time during the second period (cases 22–42) showed a significant reduction compared to the initial period (cases 1–21) (*p* = 0.004).

The mean operative duration for all RAS procedures was 132.7 min (range 38 to 502 min). This included a mean docking time of 9.8 min (range 1 to 30 min). The mean operative times in the initial period (case 1–21) and the subsequent period (case 22–42) of the study were comparable, being 134.5 ± 97.6 min and 131.0 ± 53.2 min, respectively (*p* = 0.881). Analyzing the docking times for individual cases over time revealed a decreasing trend (Figure 5). During the study, the mean docking time during the second period (cases 22–42) showed a significant reduction compared to the initial period (cases 1–21) (*p* = 0.004) (Figure 5).

None of the 42 RAS cases (0%) were converted to an open procedure during the surgical procedure. In four of the 42 cases (10%), RAS was converted to conventional laparoscopy. The reasons for conversion were suboptimal trocar positioning in three cases and a defective instrument in one case.

The procedures and detailed reasons for conversion were as follows:

(1) A bilateral inguinal hernia repair in a seven-year-old child (weight 24 kg) was converted due to the excessive force of the 3 mm instruments during docking (difficulties in setting fulcrum point);
(2) A single inguinal hernia repair in a five-year-old child (weight 20 kg) was converted due to limited motion for tissue dissection, using 5 mm instruments;
(3) A subtotal colectomy in a seventeen-year-old child (weight 55 kg) was converted due to the collision of the arms while mobilizing the splenic flexure, using 5 mm instruments;
(4) A Nissen fundoplication in an almost two-year-old child (weight 12 kg), performed using 3 mm instruments, was converted due to a defective instument.

In total, one of the 42 RAS cases experienced an intraoperative complication (2%). A three-year-old child, who initially underwent a robotic-assisted Nissen fundoplication using 3 mm instruments, required a reintervention after readmission on postoperative day three. This involved a laparoscopic exploration for a thermal gastric perforation (Clavien Grade 4 complication).

### 3.3. Postoperative Outcomes

The mean duration of the postoperative hospital stay until discharge was 3.6 days (range 0 to 46 days). The total number of postoperative complications was six out of 42 children (14%), of which three patients required reintervention (7%). Of these, two children developed four-quadrant peritonitis, on day 1 and day 3, respectively, requiring laparoscopic exploration. The first initial patient who developed peritonitis underwent a subtotal colectomy and was the same patient who was converted to conventional laparoscopy. The second initial procedure was a Ladd’s procedure with appendectomy in a four-year-old child, using 5 mm instruments. In both, no perforation was observed during re-laparoscopy, and drainage was performed along with postoperative antibiotics (Clavien Grade 3b complication). The third reintervention case involved a wound dehiscence after using a 5 mm trocar for the umbilical incision in a seven-year-old child who underwent an appendectomy. The wound was resutured at the bedside (Clavien Grade 3a complication). All patients with postoperative complications recovered completely.

Other postoperative complications included a seroma after a proctocolectomy that resolved spontaneously (Clavien Grade 1 complication), wound dehiscence after an ileocecal resection that was opened at the bedside (Clavien Grade 1 complication), and readmission for an abscess following a Ladd’s procedure and appendectomy, requiring intravenous antibiotics (Clavien Grade 2 complication). All three patients were 14–18 years old; therefore, 5 mm instruments were used during the RAS procedure.

In total, the number of readmissions was two (5%). No long-term complications occurred between 30 days and 6–8 weeks of follow-up. In this study, there were no deaths.

### 3.4. Surgeon’s Comfort

**Figure 6:**
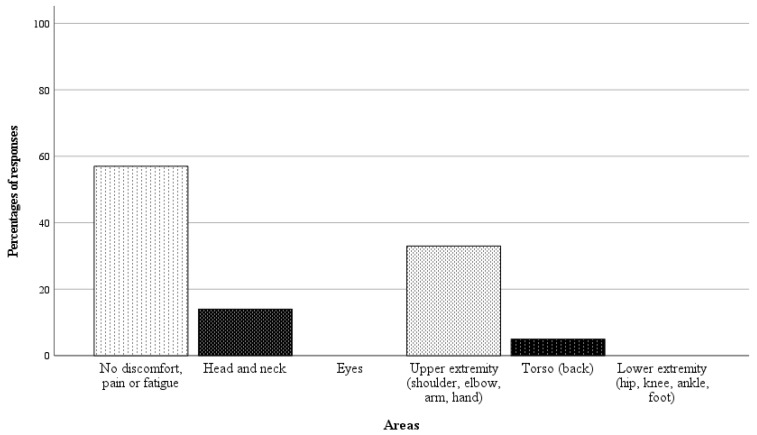
Responses as percentages (%) of the 21 completed questionnaires on surgeon’s physical demand.

**Figure 7:**
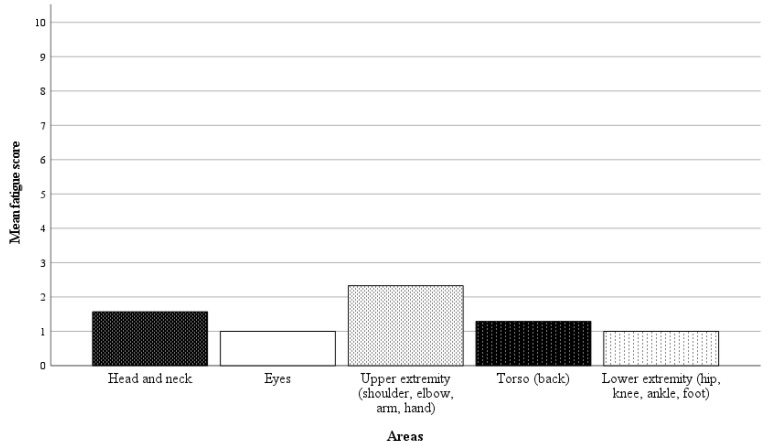
Mean fatigue scores for specific body areas (1 = no discomfort, pain, or fatigue, 10 = worst imaginable discomfort, pain, or fatigue). The average fatigue score across all body areas was 1.4 points.

In 21 of 42 randomly selected RAS cases, questionnaires on the surgeon’s physical demand were completed. Directly after performing the surgery, the surgeon was asked a binary question regarding whether there was any physical discomfort, pain, or fatigue in specific body areas, followed by a rating scale from 1 to 10 (1 = no discomfort, pain, or fatigue, 10 = worst imaginable discomfort, pain or fatigue) if the response was affirmative. The results of the analysis are presented in Figure 6 and Figure 7. In 57% of the cases, the surgeon reported no discomfort, pain, or fatigue. Among the nine cases with affirmative responses, the average fatigue score across all body areas was 1.4 points.

Overall, 90% of the RAS cases were successfully accomplished, and 86% of the children had an uncomplicated postoperative course (Table 2).

## 4. Discussion

The results of the current observational study demonstrate that the SSS^®^ is safe and feasible to use for abdominal RAS procedures in children. The success rate is high (90%), while the complication and conversion rates are low (17% and 10%, respectively). The outcomes provide new fundamental information supporting the implementation of the SSS^®^ in clinical practice in pediatric surgery.

RAS holds many theoretical advantages in pediatric surgical procedures. It inherently has benefits for patient recovery that are similar to those of laparoscopy, including minimal operative trauma leading to less postoperative pain, the limited need for postoperative opioid use, reduced hospital stays and earlier returns to school, and better cosmesis [4,5,17]. Moreover, RAS has the potential to overcome the difficulties with dexterity and the challenges of the two-dimensional view of conventional laparoscopy, which may be particularly advantageous for pediatric surgery. Even if a two-dimensional 5 mm camera is used, the quality of the image is superior because of the high-definition augmented vision and very steady images directed via the eyes of the surgeon. The design of robotic instruments that mimic human and wrist movements eases access to the smaller anatomical areas in the bodies of children [5,17,18]. Through motion scaling, the magnitude of internal instrument movement can be adapted relative to the movement of the surgeon’s hands and wrists, supporting precise movements in small and narrow spaces [5,17,18]. The visualization of the anatomy in children can be challenging given their small size. Robotic cameras provide tremor filtration, 10–15 times magnification capabilities, and operator-controlled two- or three-dimensional views, which result in the stable and precise visualization of the surgical field. In this way, RAS consequently allows for better surgeon control [5,17,18].

Nevertheless, pediatric robotic surgery has some key differences compared to adult RAS. The small body size of the child and the ever-changing proportions with age require adaptations in the robotic setup, with the unique port placement, instrument use and size, anesthesiologists’ faster access to the patient, and changes to the patient position requiring the undocking of the robot [5]. The SSS^®^ offers 3-mm-sized instruments, which makes RAS particularly suitable for younger children and newborns. The use of smaller instruments that can be placed more closely together and do not need a long insertional depth suggests that RAS might be feasible in small patients [6,11].

The results of the present study demonstrate that using the SSS^®^ in pediatric patients is safe and feasible.

The non-occurrence of conversion from RAS to an unplanned open approach during the surgical procedure shows that the SSS^®^ is feasible in pediatric cases. The rate of conversion from RAS to conventional laparoscopy is low (4 of 42 procedures, 10%) and falls within the range of an acceptable conversion rate. The overall conversion rates of initial RAS procedures in children reported in the literature are 1 to 14% [19,20,21]. We observed that the main cause of conversion was the challenge associated with the determined trocar positions, which resulted in collisions, limited motion, and force exceedance. Particularly in small children, the reduced distance between trocar placements increased the likelihood of robotic arm collisions. Children are known to have a dome-shaped abdomen, necessitating the consideration of not only the distance between the trocars but also the positioning of the robotic arms, as working from different heights increases the risk of collision. Overall, conversions primarily result from the progressive accumulation of experience in the correct positioning of the trocars and robotic arms. As RAS procedures are performed routinely with the SSS^®^, we have observed a decrease in collisions due to improved trocar positioning. The use of smaller instruments, such as 3 mm and 5 mm instruments, facilitates trocar placement because they require a shorter insertion depth. This allows the trocars to be positioned closer together, which minimizes the need for extensive extra-abdominal movement and consequently reduces the risk of collision. The importance of optimal trocar positions in children, which vary with age and body size, is a fundamental consideration in pediatric robotic cases.

The observed intraoperative and postoperative complication rates are low, reflecting the safety of RAS with the use of the SSS^®^ in children. The complication rate observed in this study is consistent with those reported in the literature on abdominal robotic surgeries in children [22]. The complications during the study period were not associated with conversion or robotic malfunctions. The safety features inherent in the SSS^®^ make the system a good choice for the performance of pediatric procedures. The unique capabilities of instruments with haptic feedback ensure the gentle handling of tissue, and forced feedback prevents the surgeon from creating accidental damage to the surrounding tissue, as this is one of the most common complications associated with robotic surgery [23]. Additionally, the required foot pedal activation before any instrument movement also contributes to this. The three individual arms can be easily undocked and moved, allowing quick access to the patient’s bedside for the surgeon (in the case of conversion) as well as the anesthesiologist and easing the repositioning of the patient during surgery [23].

While a significant concern surrounding RAS is its high costs, the SSS^®^ may be associated with lower procedural costs. The utilization of truly reusable instruments and the compatibility with existing operating room equipment (open platform) avoid additional costs. In adult gynecologic cases, the costs of the SSS^®^ appear to be equal to those of laparoscopy and less expensive compared to the DVSS^®^ [24].

Notably, the ergonomic benefit for the surgeon may also be considered when determining the worth of this investment. The SSS^®^ offers a surgical console with a comfortable, adjustable chair. In the current study, a significant proportion of the RAS cases were performed without any reported discomfort, pain, or fatigue for the surgeon. If discomfort was present, it was reported to be minimal. As demonstrated in the report presented by Menke et al., an ergonomic assessment of the SSS^®^ using the SURG-TLX workload methodology has already indicated reduced physical demands for surgeons compared to laparoscopy [25]. A comfortable ergonomic setup can improve surgeon control while operating, thereby enhancing the surgical safety.

RAS aims to enhance surgeons’ performance of the laparoscopic surgical technique with the use of the robotic system. Consequently, surgeons already experienced in laparoscopic procedures may find it relatively seamless to adopt RAS. The present study found that the mean operative times during the first period and the second period were comparable. This is likely because experienced laparoscopic surgeons do not experience significant improvements in surgery times when switching to RAS. However, in this study, the accurate assessment of the operative times was limited as different procedures were performed, and the complexity of each procedure inherently indicates the duration of the procedure. Additionally, the intervals between the performance of specific procedures could have lengthened the learning curve for each procedure. Nevertheless, RAS procedures might initially be prolonged due to the robotic setup and the learning process associated with the device’s use. The docking time of the robot did decrease over time during the study, which shows an improvement in efficiency regarding the initial setup of the robot.

The SSS^®^ offers flexibility in trocar positioning, similar to conventional laparoscopy, which facilitates conversion to laparoscopy or open surgery. An important consideration, especially in smaller children, is the pre-planning of the trocar positions to prevent collisions between the external robotic arms. The restricted internal and external operating areas in children, combined with the size and proportions changing with age, challenge the setup of the robot, which needs to be adjusted according to the procedure. Therefore, particularly in small children, besides the trocar positioning, accurate patient and table positioning is critical when performing RAS.

## 5. Conclusions

This observational cohort study confirms that using the SSS^®^ for abdominal RAS procedures in pediatric patients is both safe and feasible. RAS offers features that ease access to smaller anatomical areas, which therefore may be particularly advantageous in small children and newborns. The optimal planning of the trocar positions and patient positioning are some important aspects that should be considered when conducting RAS in pediatric cases. Based on our experience, we anticipate that the ongoing development of the SSS^®^ and our growing experience will advance the safety and effectiveness of RAS procedures in children.

## Figures and Tables

**Table 1:** The numbers of the specific robotic-assisted surgery (RAS) procedures performed, with the corresponding patient demographics.

No. Performed (*n* = 42)	Procedure	Mean Age ^1^	Mean Weight ^2^	F/M Ratio ^3^	Instrument Size ^4^
11	Nissen fundoplication	10	37.2	7/4	4 × 3 mm, 7 × 5 mm
10	Inguinal hernia repair	6.3	24.7	6/4	4 × 3 mm, 6 × 5 mm
5	Cholecystectomy	14.9	69.9	0/5	5 mm
4	Appendectomy	10.7	38.8	2/2	1 × 3 mm, 3 × 5 mm
2	Ileocecal resection	15.8	54	1/1	5 mm
2	Ladd’s procedure and appendectomy	10.9	42.5	1/1	5 mm
2	Cecostomy	14.7	41.6	1/1	1 × 3 mm, 1 × 5 mm
2	Heller–Dor procedure	11.6	36.1	1/1	1 × 3 mm, 5 mm
1	Subtotal colectomy	17.4	52	0/1	5 mm
1	Proctocolectomy	14.7	82	0/1	5 mm
1	Ileostomy	5.7	18.1	0/1	3 mm
1	Teratoma extirpation	15.2	49	0/1	5 mm

^1^ Age at time of surgery, measured in years. ^2^ Body weight at time of surgery, recorded in kilograms. ^3^ M = male, F = female. ^4^ Size of the (working) instruments of the Senhance^®^ Surgical System used during the procedure.

**Table 2:** Characteristics and intra- and postoperative outcomes of pediatric patients who underwent robotic-assisted surgery (RAS) using the Senhance^®^ Surgical System (*n* = 42).

	RAS Procedures (n = 42)	Mean Difference (95% IC)	*p*-Value ^1^
**Clinical characteristics**			
Sex in no. (%)			
Male	20 (48)		
Female	22 (52)		
Age at time of surgery, mean in y (SD)	10.7 (±5.5)		
Body weight at time of surgery, mean in kg (SD)	40.7 (±24.1)		
**Intraoperative**			
Conversion to conventional laparoscopy, no. (%)	4 (10)		
Conversion to open procedure, no. (%)	0 (0)		
Operative time ^2^, mean in minutes (SD)			
Total study time	132.7 (±77.7)		
Period 1 (case 1–21)	134.5 (±97.6)		
Period 2 (case 22–42)	131.0 (±53.2)	3.5 (−44.3–51.2)	0.881
Docking time of robot ^3^, mean in minutes (SD)	9.8 (±7.0)		
Period 1 (case 1–21)	13.1 (±7.9)		
Period 2 (case 22–42)	6.5 (±3.8)	6.6 (2.3–10.8)	0.004
Intraoperative complications, no. (%)	1 (2)		
**Postoperative**			
Postoperative hospital stays ^4^, mean in days (SD)	3.6 (±7.5)		
Postoperative complications ^5^, no. (%)	6 (14)		
Long-term complications ^6^, no. (%)	0 (0)		
Readmission through 30 days, no. (%)	2 (5)		
Reintervention, no. (%)	3 (7)		
Mortality, no. (%)	0 (0)		
Clavien–Dindo Classification for complications [15], no.			
1	2		
2	1		
3a	2		
3b	1		
4a	1		
4b	0		
5	0		
Total number of complications, no. (%)	7 (17)		

^1^ For continuous variables, either a paired *t*-test or Wilcoxon signed-rank test was used as appropriate. Nominal data were analyzed using the McNemar test. A *p*-value of less than 0.05 was deemed statistically significant. ^2^ Operative time refers to the duration of the surgery, from the initial skin incision to the final wound closure. ^3^ Docking time is defined as the period required to position the robot and ensure that the instruments are correctly aligned. ^4^ Postoperative hospital stay is calculated based on the number of nights spent in the hospital after the surgery (i.e., discharge on the same day is considered 0 nights, while discharge the next day counts as 1 night). ^5^ Postoperative complications are defined as any issues that arise within 30 days following the surgery. ^6^ Complications that occur between 30 days and 6–8 weeks after the operation are also recorded [13]

## Data Availability

All data are stored securely at the Department of Pediatric Surgery of MosaKids Children’s Hospital/Maastricht University Medical Center+ (MUMC+) without patient identifiers and are available for inspection upon request.
